# Liver Lobe Based Multi-Echo Gradient Recalled Echo T_2_*-Weighted Imaging in Chronic Hepatitis B-Related Cirrhosis: Association with the Presence and Child-Pugh Class of Cirrhosis

**DOI:** 10.1371/journal.pone.0154545

**Published:** 2016-05-12

**Authors:** Dan Wang, Tian-wu Chen, Xiao-ming Zhang, Jie Li, Nan-lin Zeng, Li Li, Yu-lian Tang, Yu-cheng Huang, Rui Li, Fan Chen, Yan-li Chen

**Affiliations:** 1 Sichuan Key Laboratory of Medical Imaging, and Department of Radiology, Affiliated Hospital of North Sichuan Medical College, Nanchong, Sichuan 637000, China; 2 Department of Pathology, Affiliated Hospital of North Sichuan Medical College, Nanchong, Sichuan 637000, China; Taipei Veterans General Hospital, TAIWAN

## Abstract

**Purpose:**

To investigate whether liver lobe based T_2_* values measured on gradient recalled echo T_2_*-weighted imaging are associated with the presence and Child-Pugh class of hepatitis B-related cirrhosis.

**Methods:**

Fifty-six patients with hepatitis B-related cirrhosis and 23 healthy control individuals were enrolled in this study and underwent upper abdominal T_2_*-weighted magnetic resonance imaging. T_2_* values of the left lateral lobe (LLL), left medial lobe (LML), right lobe (RL) and caudate lobe (CL) were measured on T_2_*-weighted imaging. Statistical analyses were performed to determine the association between liver lobe based T_2_* values and the presence and Child-Pugh class of cirrhosis.

**Results:**

The T_2_* values of the LLL, LML and RL decreased with the progression of cirrhosis from Child-Pugh class A to C (r = -0.231, -0.223, and -0.395, respectively; all *P* < 0.05), except that of the CL (r = -0.181, *P* > 0.05). To a certain extent, Mann-Whitney U tests with Bonferroni correction for multigroup comparisons showed that the T_2_* values of the LLL, LML and RL could distinguish cirrhotic liver from healthy liver (all *P* < 0.05), whereas the T_2_* values of the CL could not (*P* > 0.05). Receiver operating characteristic analysis demonstrated that the T_2_* value of the RL could best distinguish cirrhosis from healthy liver, with an area under the receiver operating characteristic curve (AUC) of 0.713 among T_2_* values of the liver lobes, and that only the T_2_* value of the RL could distinguish Child-Pugh class C from A-B, with an AUC of 0.697 (all *P* < 0.05).

**Conclusion:**

The T_2_* value of the RL can be associated with the presence and Child-Pugh class of hepatitis B-related cirrhosis.

## Introduction

Liver cirrhosis is a frequent consequence of chronic liver diseases, such as Hepatitis B, and is characterized by tissue fibrosis and the conversion of healthy liver architecture into structurally abnormal nodules [1]. Assessing the presence and severity of cirrhosis is crucial when selecting therapeutic approaches and monitoring patient responses to clinical interventions. Although a liver biopsy is the standard assessment, it has several disadvantages, including invasiveness, complications, interobserver variability, and sampling errors [2–4]. Following noninvasive procedures to assess liver function, the Child-Pugh classification system divides patients with cirrhosis into low (class A), intermediate (class B), and poor (class C) risk categories to differentiate between the least sick and the most advanced patients [5].

As cirrhosis progresses, liver iron deposition increases, and hemodynamic disorder occurs in advanced cirrhosis [6, 7]. As a noninvasive procedure, magnetic resonance imaging(MRI) has been used to assess liver cirrhosis, and non-contrast-enhanced multi-echo gradient recalled echo (GRE) T_2_*-weighted imaging (T_2_*WI) has been regarded as an important method to accurately measure iron load and deoxyhemoglobin in the liver using the T_2_* value [3,8–10]. According to Chung et al. [11], the T_2_* value of a cirrhotic liver is lower than that of a healthy liver and decreases as the Child-Pugh class progresses from A to C, due to increasing liver iron concentrations. As reported by Jin et al. [12], when deoxyhemoglobin increases in cirrhotic livers, the T_2_* value is lower than that of healthy livers and decreases with the severity of liver fibrosis. The previous findings were based on the T_2_* value of the whole liver.

However, hypertrophy of the caudate lobe (CL) and left lateral lobe (LLL) as well as atrophy of the right lobe (RL) and left medial lobe (LML) occur as the liver progresses from being healthy to having compensatory cirrhosis [13, 14]. In this process, we hypothesize that the iron or deoxyhemoglobin concentrations increase in each liver lobe at different rates and considered which liver lobe T_2_* value could be best associated with the presence and Child-Pugh class of cirrhosis. To the best of our knowledge, no reports have focused on the utility of liver lobe based T_2_* values to assess liver cirrhosis. Therefore, the aim of this study was to investigate how the liver lobe based T_2_* values could be associated with liver cirrhosis and to determine which lobe parameter could be the best indicator to determine the presence of cirrhosis and Child-Pugh class.

## Materials and Methods

### Patients

This prospective study was approved by the institutional ethics review board of North Sichuan Medical College, and a written informed consent was obtained from each participant prior to the study.

From February 2013 to October 2014, 61 consecutive patients undergoing abdominal MRI were enrolled into our study according to the following inclusion criteria: (1) a diagnosis of cirrhosis in patients with hepatitis B based on histopathological findings and laboratory investigations combined with image findings whenever available, according to the American Association for the Study of Liver Diseases (AASLD) practice guidelines on chronic hepatitis B (2007) [15]; (2) a Child-Pugh score calculation, performed using 5 parameters including albumin, ascites, bilirubin, prothrombin activity and encephalopathy; and (3) MRI data that showed cirrhotic patients without hepatic carcinoma, hepatic hemangioma, or other malignant or benign tumors. In this cohort, 46 cases with cirrhosis were diagnosed as fibrosis stage 4 according to the METAVIR classification system as shown on liver biopsy by an experienced, associated professor (the sixth author, with 14 years of experience in pathology); the remaining 15 cases were not diagnosed by liver biopsy but were based on clinical, biological, endoscopic and MRI data according to the previous AASLD guidelines because of coagulation dysfunction.

The exclusion criteria were as follows: (1) patients had a history of treatment for portal hypertension, such as a splenectomy or endoscopic therapies before MRI and biochemical tests (n = 2); (2) patients had active alcohol abuse (n = 1); or (3) patients had primary biliary cirrhosis (n = 2). Consequently, 56 patients were included in this study. In this cohort, there were 37 men and 19 women, ranging from 26 to 80 years of age (median age, 54.5 years). According to the Child-Pugh classification system, 17, 22 and 17 patients were categorized into Child-Pugh class A, B and C, respectively. The Child-Pugh score was calculated within one week after the patient received the MRI. In addition, the enrolled patients underwent medical or surgical treatments for portal hypertension after MR imaging, and biochemical tests were performed for a definite diagnosis.

During the same research period, 23 participants with no history of chronic liver disease (10 men and 13 women; mean age, 50 years; range 29–74 years) served as a control group. All participants underwent upper abdominal MRI scans including GRE-T_2_*WI for an adrenal tumor (n = 8), splenic cyst (n = 5), renal hemangioma (n = 6), or retroperitoneal lipoma (n = 4).

### MRI technique

Abdominal MR examination was performed using a 3.0-T scanner (Discovery MR 750; GE Medical Systems, Milwaukee, WI) in a 32-channel phased array body coil after the respiratory signals were established. All participants underwent routine MRI including axial liver acquisition with volume acceleration flex (LAVA-Flex) MR imaging, fat-suppressed propeller T_2_-weighted imaging, and GRE T_2_*-weighted fat-suppressed sequence followed by the enhanced MR sequences. The T_2_*WI of the liver was obtained using a single 6-mm slice through the center of the liver at 16 different echo times. The T_2_*WI was acquired during a breath-hold using a gradient-echo sequence according to the following scanning parameters: repetition time of 57.4 ms, echo time of 1.3–32 ms, flip angle of 25°, acquisition matrix of 256×192, pixel size of 1.6×1.6 mm, field of view of 420×360 mm, and sampling bandwidth of 83.3 kHz. The T_2_*WI lasted a total of 55 s consisting of three 15-s breath-holds each followed by a 5-s free breathing until the end of the third breath-hold, and the T_2_*WI was performed during each breath-hold and was stopped during each free breathing. Subsequently, gadodiamide (Magnevist; Bayer Healthcare, Germany) was intravenously injected via a pressure injector (Spectris MR Injection System; Medrad, Warrendale, PA) according to 0.2 mmol/kg body weight at a rate of 3 mL/s followed by a 20-mL saline solution flush for axial contrast enhanced three-dimensional LAVA. In addition, LAVA-Flex MR imaging, fat-suppressed propeller T_2_-weighted imaging and enhanced MRI were performed to identify whether the cirrhotic patients could be included in the research cohort and to depict the adrenal tumor, splenic cyst, renal hemangioma or retroperitoneal lipoma in the control group.

### Image data analysis

The prospective original MRI data were directly interfaced and forwarded to a workstation (GE Advanced Workstation version 4.4–09, Sun Microsystems, Palo Alto, CA). As depicted in the Goldsmith and Woodburne system [16], the liver was divided into four lobes: left lateral lobe (LLL), left medial lobe (LML), right lobe (RL) and caudate lobe (CL). The T_2_* values of each liver lobe were retrospectively and independently measured by R2star Map on the MRI workstation by two radiologists (the first author, with 3 years of radiology experience; and the corresponding author, with 17 years of abdominal MRI experience) who were blinded to the clinical data and pathology reports.

**Fig 1:**
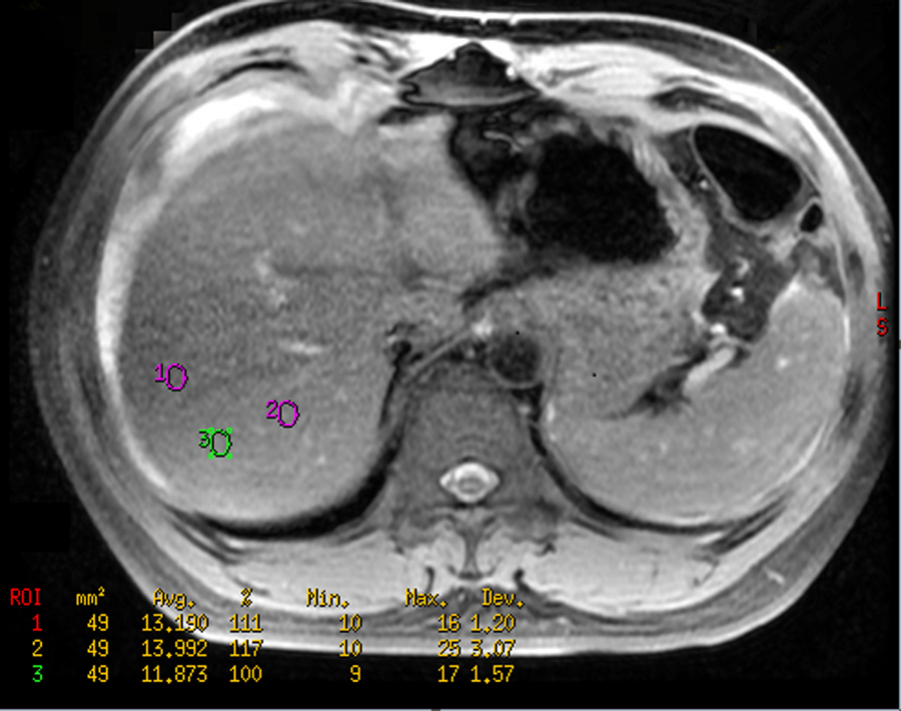
The outlines of regions of interest to obtain the liver lobe based T2* values. In a 51-year-old male with Child-Pugh Class C of liver cirrhosis, three regions of interest are randomly drawn in right liver lobe in a representative axial plane by the software to obtain the T_2_* value of this liver lobe, and the similar methods are used to obtain the T_2_* values of the other liver lobes.

We placed 3 regions of interest (ROIs, measuring 49–60 mm^2^) on a representative axial section of the LLL, LML or RL while 2 ROIs were placed on a representative axial plane of the CL because the volume of the CL was smaller than the other liver lobes (Fig 1). The previous process was repeated on three contiguous, representative, transverse sections of each liver lobe, totaling 9 ROIs in the LLL, LML or RL and 6 ROIs in the CL per participant. All ROIs were sketched to avoid any vessels and bile ducts within a distance of 1 cm from the liver capsule to reduce the effect of susceptibility artifacts from adjacent structures or intestinal gas. The T_2_* values of each liver lobe were averaged across all the corresponding ROIs in a section, and the final mean liver lobe based T_2_* value was obtained by averaging across the 3 representative sections. To verify the intra-observer reproducibility of the liver lobe based T_2_* value measurement, all T_2_* value measurements of each liver lobe were repeated 1 month later by the first author.

### Statistical analysis

SPSS statistical package version 13.0 (Chicago, IL) was used for statistical analysis. A value of *P* < 0.05 was considered to represent a significant difference. Descriptive statistics included median with 25%-75% interquartile range of liver lobe based T_2_* value, except for the mean and standard deviation (SD) of age and body weight. T_2_* values of each liver lobe were compared among Child-Pugh classes of cirrhosis using Mann-Whitney U tests with Bonferroni correction for multigroup comparisons because the data distribution was skewed. The correlation between the T_2_* value of each liver lobe and the Child-Pugh class of cirrhosis was performed using the Spearman’s rank correlation coefficient. If the T_2_* values of any liver lobe significantly differed between any two Child-Pugh classes, a receiver operating characteristic (ROC) analysis was subsequently performed to determine how the T_2_* values of this lobe could help identify the presence of cirrhosis and Child-Pugh class.

In addition, the cirrhotic patients were randomly chosen to determine the reproducibility of the measurements of liver lobe based T_2_* value. The interobserver agreement between the two independent observers’ measurements of liver lobe based T_2_* values and the intra-observer agreement between the two sessions of the first author’s measurements of the T_2_* values were assessed using coefficient of variation (CV) according to the following formula: CV = SD/mean × 100 [17]. When the coefficient of variation was less than 10%, the agreements in the inter- or intra-observers’ measurements were regarded as small, and the liver lobe based T_2_* values of the first author’s first measurement were used as the final results to perform further analysis. When the coefficient of variation was more than 10%, two additional measurements were performed by the two authors, and an average of the four measurements was used as the final liver lobe based T_2_* value.

## Results

### Inter- and intra-observer variability of the liver lobe based T_2_* value measurements

In the enrolled cirrhotic patients, the inter- or intra-observer mean CV of liver lobe based T_2_* value is shown in Table 1. The number of patients with coefficients of variation less than 10% and exceeding 10% are also shown in Table 1. When the inter- or intra-observer coefficients of variation exceeded 10%, two additional measurements were conducted by the two observers, and an average of the four measurements was used as the final liver lobe based T_2_* value.

**Table 1:** Interobserver and intraobserver variability of liver lobe based T2* value in cirrhotic patients.

Liver lobe based T_2_* value		Mean coefficient of variation (range)	≤ 10% (n)	> 10% (n)
Interobserver				
	RL	5.3 (1%-22%)	47	9
	LML	7.3 (1%-18%)	46	10
	LLL	7.1 (1%-16%)	49	7
	CL	7.5 (2%-12%)	50	6
Intraobserver				
	RL	4.7 (1%-16%)	51	5
	LML	6.5 (1%-15%)	47	9
	LLL	6.2 (1%-14%)	49	7
	CL	7.0 (1%-11%)	48	8

RL = Right liver lobe, LML = Left medial liver, LLL = Left lateral liver lobe, and CL = Caudate lobe.

### Associations of liver lobe based T_2_* value and possible clinical variables with the presence of cirrhosis and Child-Pugh class

The clinical data including gender, age, body weight and liver lobe based T_2_* values of all participants are shown in Table 2. The univariate analysis showed that the cirrhotic patients were more likely to have lower T_2_* values of the LLL (*P* = 0.036), LML (*P* = 0.046), RL (*P* = 0.004), and CL (*P* = 0.151) than the control participants. There were no significant differences in gender (*P* = 0.65), age (*P* = 0.11), or body weight (*P* = 0.77) between the cirrhotic patients and the control participants. Spearman’s rank correlation analysis illustrated that there was a trend toward decreasing T_2_* values of the LLL (r = -0.231, *P* = 0.041), LML (r = -0.223, *P* = 0.048), RL (r = -0.395, *P* < 0.001), and CL (r = -0.181, *P* = 0.11) with increasing Child-Pugh class of cirrhosis in our study.

**Table 2:** Main parameters of the healthy participants and patients with cirrhosis in different Child-Pugh classes.

Parameters	No cirrhosis	Child-Pugh class of cirrhosis			
	(n = 23)	Class A (n = 17)	Class B (n = 22)	Class C (n = 17)	Class A-B (n = 39)
Gender (M/F)	10/13	11/6	14/8	12/5	25/14
Age	49.91 ±12.91	54.52 ±12.62	55.36 ±10.86	54.71 ±11.47	54.43 ±11.50
Body weight (kg)	57.69 ±7.33	60.94 ± 4.62	57.5 ±5.36	55.0 ±4.55	59 ±5.28
LLL T_2_* (ms)	14.85	13.59	13.83	12.91	13.60
	(13.15–16.44) ^a^	(12.54–14.16)	(12.39–14.95)	(10.68–14.75)	(12.47–14.63)
LML T_2_* (ms)	15.19	14.02	14.02	13.43	14.02
	(13.12–17.83) ^a^	(12.58–14.89)	(12.99–16.36)	(10.70–15.12)	(12.59–15.30)
RL T_2_* (ms)	15.85	14.22	13.58	12.53	14.76
	(13.99–16.56) ^a^	(13.21–15.18)	(12.94–15.89)	(10.88–14.22) ^b^	(13.56–16.45) ^c^
CL T_2_* (ms)	15.08	14.32	14.40	13.89	14.32
	(13.54–16.44)	(12.38–17.27)	(12.41–16.91)	(10.71–16.23)	(12.45–16.78)

LLL T_2_* = T_2_* value of left lateral liver lobe, LML T_2_* = T_2_* value of left medial liver lobe, RL T_2_* = T_2_* value of right liver lobe, and CL T_2_* = T_2_* value of caudate lobe.

^a^ different from cirrhotic patient group, *P* < 0.05

^b^ different from participants without cirrhosis, *P* < 0.05; and

^c^ different from Class C, *P* < 0.05.

The figure in the bracket is 25%-75% interquartile range of the corresponding liver lobe based T_2_* value.

As shown by the Mann-Whitney U tests with Bonferroni correction for multigroup comparisons to test for the differences in liver lobe based T_2_* values between normal livers and each Child-Pugh cirrhosis class (Table 2), the T_2_* value of the RL was significantly lower in Child-Pugh Class C livers than in normal livers (*P* = 0.001) while no significant difference could be found in the T_2_* value of any other liver lobe (all *P* > 0.05). No significant differences were found when comparing the liver lobe based T_2_* value between participants stratified by Child-Pugh class.

Because patients with Child-Pugh Class A and B cirrhosis have a better prognosis than patients with Child-Pugh Class C [18], patients in Class A and B were combined into one group for further statistical analysis. A Mann-Whitney U test (Table 2) showed that the T_2_* value of the RL could distinguish Class A-B from C (*P* = 0.02) but the T_2_* value of any other liver lobe could not (all *P* > 0.05).

### ROC analysis of liver lobe based T_2_* value for determining the presence of cirrhosis and Child-Pugh class

**Fig 2:**
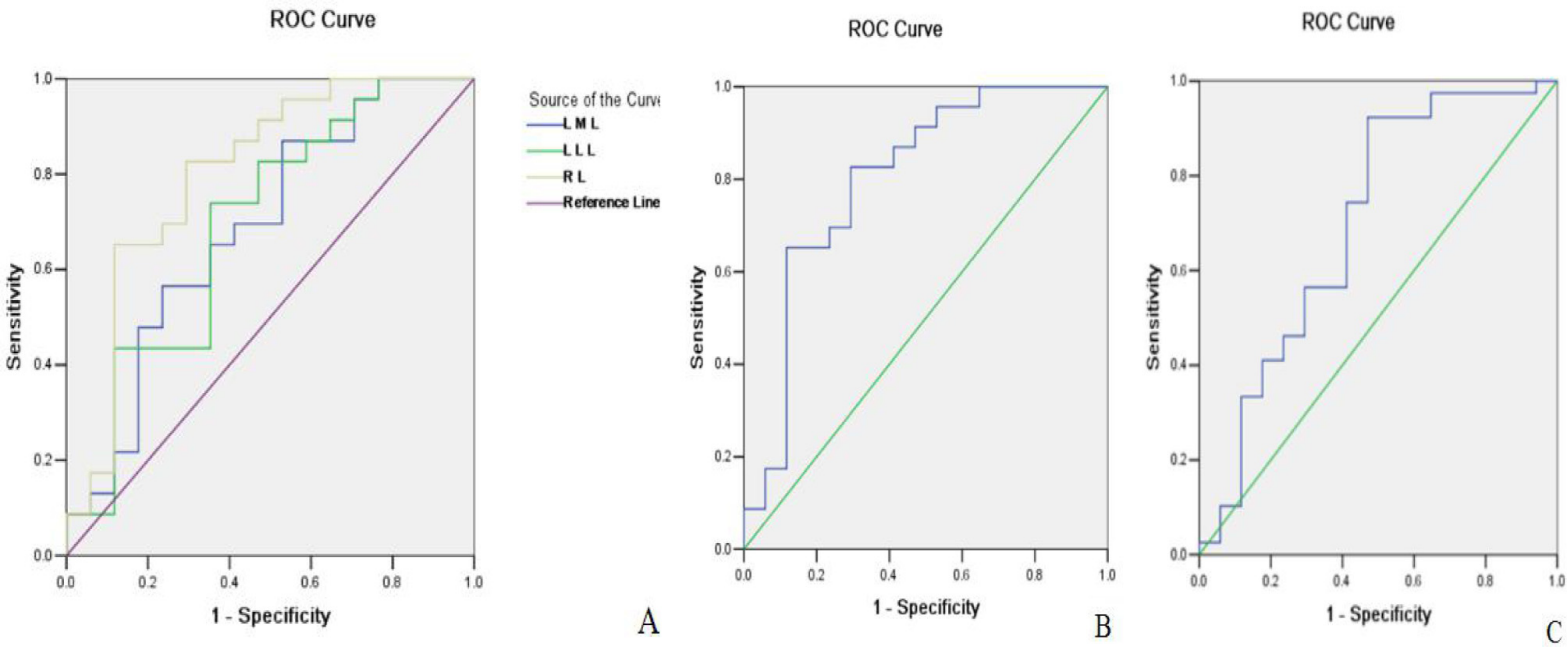
Receiver operating characteristic curves of liver lobe based T2* values to identify the presence and Child-Pugh class of cirrhosis in patients with hepatitis B. The figures show that T_2_* value of left medial liver lobe (LML), left lateral liver lobe (LLL), and right liver lobe (RL) are indicators for the discrimination between cirrhosis patients and healthy participants (A), and between Class C and healthy participants (B), and between Child-Pugh class A-B and C of cirrhosis (C).

Because there were significant differences in the T_2_* values of the LLL, LML and RL between cirrhotic patients and control participants and in the T_2_* value of the RL between Child-Pugh Class C cirrhosis and normal liver or Child-Pugh Class A-B of cirrhosis, a ROC analysis was performed to determine how to use the previous parameters to identify the presence of cirrhosis and the Child-Pugh class. The cutoff value of liver lobe based T_2_* value, area under receiver operating characteristic curve (AUC), and sensitivity and specificity are shown in Table 3. As demonstrated in Fig 2A, the T_2_* value of the RL was the best parameter at discriminating between cirrhosis patients and control participants. Only the T_2_* value of the RL could distinguish Child-Pugh Class C of cirrhosis from the control participants (Fig 2B) and Child-Pugh Class A-B from Class C (Fig 2C).

**Table 3:** Liver lobe based T2* value in determining the presence and Child-Pugh class of cirrhosis.

Parameters	Cut-off	Differentiations	AUC	Sensitivity	Specificity
LMLT_2_* (ms)	13.605	N vs. cirrhosis	0.644	73.9%	55.4%
LLL T_2_* (ms)	14.945	N vs. cirrhosis	0.660	60.9%	69.6%
RL T_2_* (ms)	14.915	N vs. cirrhosis	0.713	65.2%	76.8%
	14.81	N vs. Class C	0.801	65.2%	88.2%
	12.58	Class A-B vs. C	0.697	92.3%	52.9%

LLL T_2_* = T_2_* value of left lateral liver lobe, LML T_2_* = T_2_* value of left medial liver lobe, RL T_2_* = T_2_* value of right liver lobe, AUC = area under the receiver operating curve, and N = no cirrhosis.

## Discussion

Iron is an essential metal for humans because it is a critical element constituting hemoglobin and is involved in oxidation reactions and cell proliferation. In the human body, iron is stored in ferritin, which also has been used as a diagnostic indicator of liver iron concentrations [19]. However, serum ferritin levels can be confounded by factors such as infection, inflammation and malignancy, which were avoided when the participants were enrolled in our study. Because T_2_*WI has been regarded as a more accurate method for measuring iron loads than serum ferritin levels in cirrhotic livers, T_2_*WI was performed in our study to detect iron overload in livers. Moreover, the measurement of liver lobe based T_2_* values may be a reproducible technique to evaluate iron loads and deoxygenated hemoglobin levels because satisfactory inter- and intra-observer agreement were obtained from most of the cirrhotic patients in this study. Although the coefficient of variation was more than 10% in a few patients in our study, two additional measurements were performed and an average of the four measurements was used as the final liver lobe based T_2_* value to achieve a relatively high degree of agreement.

As shown in our study, T_2_* values of the LLL, LML and RL in patients with cirrhosis could be lower than in the livers of healthy patients and would decrease with the progress of the Child-Pugh class of cirrhosis. The following two mechanisms are expected to explain this finding. The first mechanism is iron overload in the cirrhotic liver. Iron-generated oxyradicals contribute to the peroxidation of lipid membranes, leading to organelle fragility and cellular toxicity, and finally resulting in hepatocellular necrosis and/or apoptosis with the subsequent activation of hepatic stellate cells and the development of hepatic fibrosis and cirrhosis [20]. In cirrhotic patients, iron overload increases in the liver and shortens the T_2_* relaxation time with its paramagnetism, which can explain why the T_2_* value of a cirrhotic liver would be lower than that of a healthy liver. With the increase of Child-Pugh class of cirrhosis, iron overload increases markedly in the liver, and the T_2_* values decrease accordingly.

Second, deoxygenated hemoglobin resulting from hemodynamic disorders can be another important mechanism. In patients with cirrhosis, liver hemodynamic disorders occur [7]. Blood flow to the liver is unique due to the dual supply from the portal vein and the hepatic artery, and 75%-80% of the blood entering the liver is partially deoxygenated venous blood supplied by the portal vein [21]. When hepatic fibrosis and cirrhosis occur, portal venous blood flow progressively decreases and bypasses the liver parenchyma via portosystemic venous shunts [12, 22]. There remains a lack of oxygen and microcirculation disturbance in the liver tissue even though the blood flow of hepatic artery increases to counteract the effect of the reduced portal venous blood flow. Under this circumstance, deoxygenated hemoglobin (a paramagnetic substance) increases in cirrhotic livers compared with healthy livers and increases with the progress of Child-Pugh class. As a result, the T_2_* value of a cirrhotic liver can be lower than a healthy liver and can decrease as cirrhosis progresses.

As shown in our study, the T_2_* value of the RL could decrease more obviously than that of the LLL, LML, and CL and can distinguish Class A-B from Class C of cirrhosis and Class C from healthy livers. This finding can be explained by the anatomy of the portal venous system. The right portal vein branch enters directly into the parenchyma of right lobe [23]. In cirrhotic livers, hepatic fibrosis and regenerative nodules cause compression and irregular stenoses of the intrahepatic branches of the right portal vein and reduce the blood flow through this portal vein, resulting in serious oxygen deficits in this liver lobe [24]. However, the left portal vein runs through the falciform ligament before entering the left liver lobe [25], leading to a relatively greater blood flow for this liver lobe. Under this circumstance, the oxygen deficit in the left lobe might be less than in the right lobe, resulting in less deoxygenated hemoglobin in the left lobe than in the right lobe. Therefore, the T_2_* value of the right lobe could decrease more obviously than that of the left lobe.

In addition, our study showed that there were no significant differences in the T_2_* values of the CL in all comparisons, but a slight downward trend of T_2_* values of the CL may be found with increasing Child-Pugh cirrhosis classes. The mechanism for explaining this finding is the blood supply of the caudate lobe arising from the bifurcation of the portal vein, which has a shorter intrahepatic course [25]. Thus, in cirrhotic patients, the hemodynamic disorder in the caudate lobe is not more serious than in any other liver lobe.

As shown by ROC analysis, the T_2_* values of the LLL, LML and RL could distinguish cirrhotic livers from healthy livers. Among these parameters, the T_2_* value of the RL was the best parameter to discriminate between cirrhotic and healthy livers. Furthermore, our study illustrated that the T_2_* value of the RL was the only liver lobe based T_2_* value to distinguish Class A-B from Class C cirrhosis. Therefore, the T_2_* value of the RL could be a suitable tool to identify the occurrence and Child-Pugh class of cirrhosis.

Compared with previously published papers, our study has several advantages. First, we used a 3.0-T scanner instead of a 1.5-T MR scanner for this study. The liver lobe based T_2_* value obtained with a 3.0-T scanner can be more accurate than with a 1.5-T scanner, and the effect of shortening the T_2_* relax time by deoxygenated hemoglobin can be clearly observed in high tesla MRI [26, 27], which results in liver lobe based T_2_* values in cirrhotic and healthy livers that are lower than those reported in other published studies. Second, we used the liver lobe based T_2_* values to assess liver cirrhosis and determined the association of the liver lobe based T_2_* values with the presence and Child-Pugh class of cirrhosis. Our study suggested that the T_2_* value of the RL can be recommended as a suitable parameter for identifying the occurrence and Child-Pugh class of cirrhosis.

Our study has a limitation. The sample size was relatively small, but our study provided some useful information about the association of the liver lobe based T_2_* values with the presence and Child-Pugh class of cirrhosis. A large scale study will be performed in our future studies to confirm the results.

In conclusion, we used liver lobe based T_2_* values to assess cirrhosis. Liver lobe based T_2_* values could decrease from healthy to cirrhotic livers and with the progression of Child-Pugh class of cirrhosis. Moreover, the T_2_* value of the RL can be recommended as a suitable parameter to identify the occurrence and Child-Pugh class of cirrhosis. These findings could be helpful to select the appropriate liver lobe based T_2_* value to identify the presence and severity of liver cirrhosis in clinical settings.
